# Supplementary material for: Interplay between MPIase, YidC, and PMF during Sec-independent insertion of membrane proteins
Source: Life Sci Alliance. 2021 Oct 12;5(1):e202101162. doi: 10.26508/lsa.202101162 (PMC8548208; doi:10.26508/lsa.202101162)
Supplement: Supplementary file 1 [file LSA-2021-01162_SdataF1.pdf]

Fig. 1B

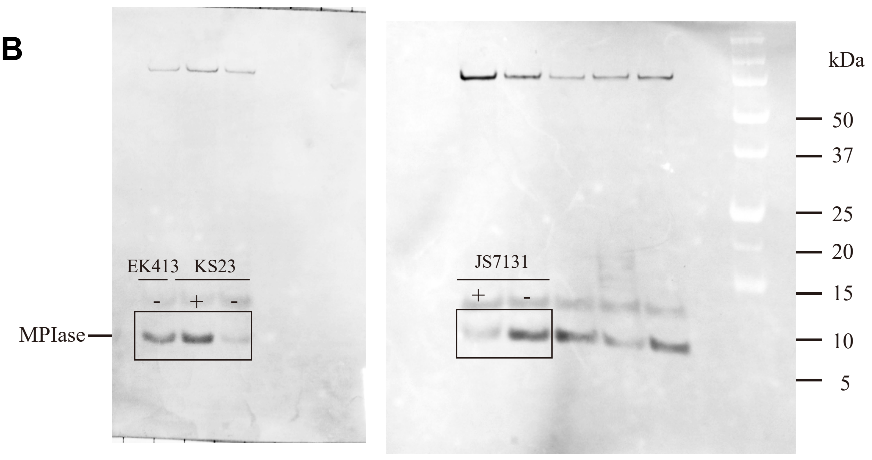

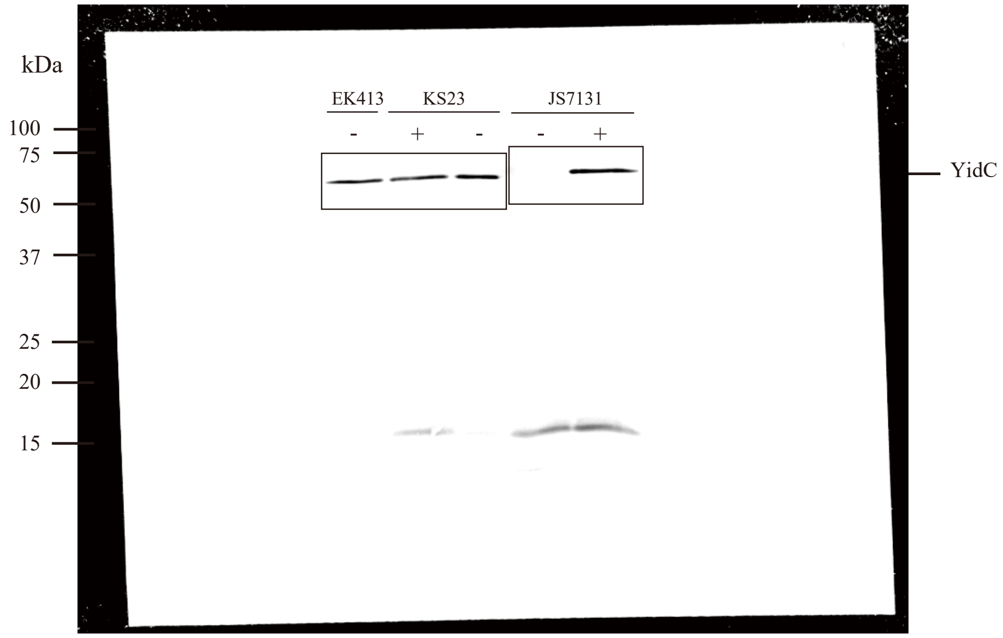

Fig. 1D

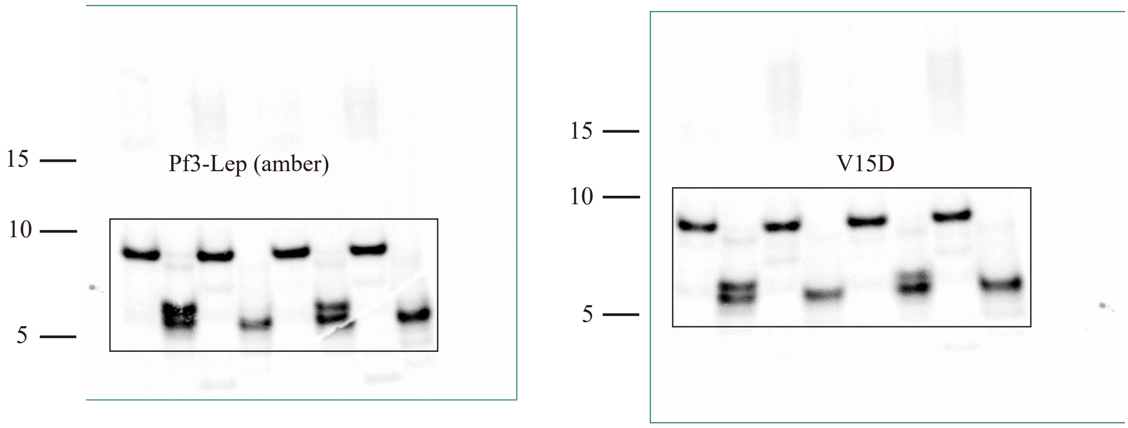

Source data for Fig. 1B and 1D. Used regions are boxed. Essentially, no bands were observed outside the boxed regions of all the autoradiograms.
