# Supplementary figures and images for: Interplay between MPIase, YidC, and PMF during Sec-independent insertion of membrane proteins
Source: Life Sci Alliance. 2021 Oct 12;5(1):e202101162. doi: 10.26508/lsa.202101162 (PMC8548208; doi:10.26508/lsa.202101162)

**Fig. 5A**

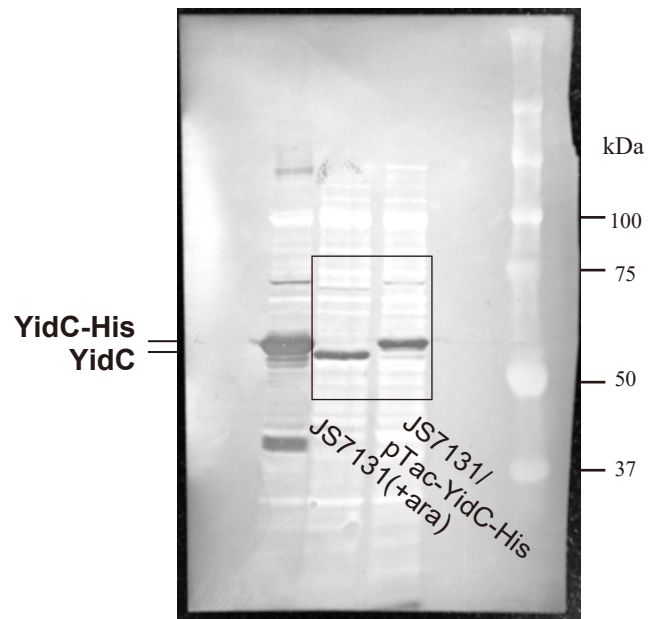

**Fig. 5B**

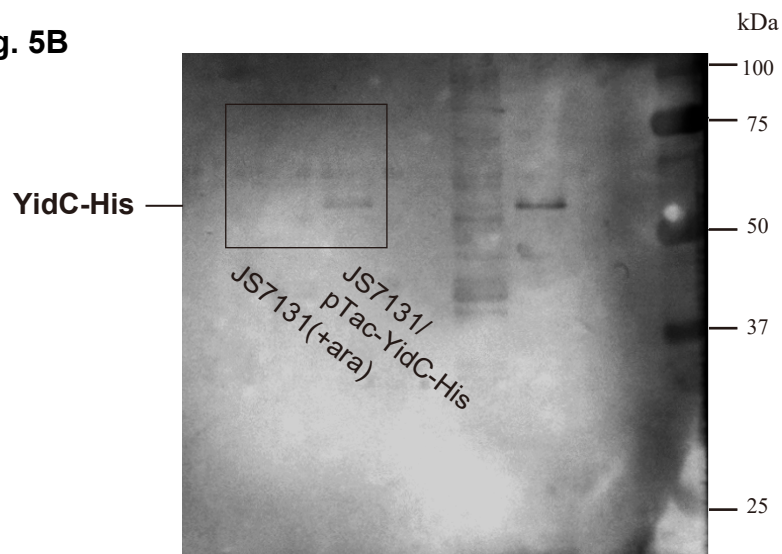

Source data for Fig. 5A and 5B. Used regions are boxed.

Supplement: Supplementary file 2 [file LSA-2021-01162_SdataF5.pdf]
